# Supplementary material for: The genome sequence of Carex divulsa Stokes, 1787 (Poales: Cyperaceae)
Source: Wellcome Open Res. 2026 Jun 25;11:381. [Version 1] doi: 10.12688/wellcomeopenres.26811.1 (PMC13755620; doi:10.12688/wellcomeopenres.26811.1)
Supplement: Peer review history

# Reviewer report: Reviewer response for version 1

**DOI:** 10.21956/wellcomeopenres.29522.r179027

**Published:** 2026-11-11

**Authors:** Ruslan Kalendar

**Affiliations:** 1 University of Helsinki, Helsinki, Finland

The authors present a chromosome-level reference assembly (lpCarDivu1.1) of Carex divulsa (Grey Sedge), produced through the Darwin Tree of Life pipeline from a single leaf specimen collected in Kingston upon Thames, UK.

 PacBio HiFi reads (23.13 Gb, about 49× of the flow-cytometry estimate) were assembled with Hifiasm, scaffolded with Arima Hi-C data in YaHS and manually curated. The primary assembly spans 388.30 Mb in 35 scaffolds (contig N50 2.9 Mb, scaffold N50 13.44 Mb), with 99.95% of the sequence placed in 27 chromosomal pseudomolecules.

 Consensus accuracy was very high (QV 70.7) and k-mer completeness was 99.17%, but BUSCO completeness against poales_odb10 was only 75.5%. Two mitochondrial sequences (1,043.83 and 330.03 kb) and a 183.84 kb plastome are also provided. All raw data and assemblies are publicly available under ENA BioProject PRJEB68040.

**Overall assessment**

 The data are of high quality, were generated with the established and well-documented Darwin Tree of Life workflow and are fully public; therefore, the note fulfils its purpose as a data release.

 My comments concern reporting rather than the data. Three issues deserve explicit discussion in the text: the 60-80 Mb gap between assembly size and the independent genome size estimates; a BUSCO result that falls short of the EBP benchmark without comment; and the mismatch between 27 pseudomolecules and the published chromosome counts for the species (2n = 56-58).

 Addressing these issues, together with the internal inconsistencies listed under minor comments, would make the manuscript considerably more useful to readers working on Carex genome and karyotype evolution research. I agree with the first reviewer that a preliminary annotation would add value, but I do not consider it essential for a Data Note.

 1. The assembly (388.30 Mb) is 82.6% of the flow cytometry estimate (470 Mb) and 86.3% of the GenomeScope2 estimate (449.78 Mb); therefore, approximately 60-80 Mb is not represented. The high k-mer completeness (99.17%) does not contradict this, because collapsed repeats reduce copy number rather than k-mer presence.

 2. The mean HiFi read length is only about 8.8 kb (23.13 Gb from 2.62 million reads), and the sheared DNA (10.7 kb, GQN 4.9) fell below the 12-20 kb target. 

 3. BUSCO gives C 75.5% (S 68.2%, D 7.3%) with 22.3% missing, which fails the targets listed in Table 4 (S > 90%, D < 5%). The text nevertheless states only that the assembly meets the 6.C.Q40 standard. 

 The low score is very likely a property of the reference set rather than the assembly. poales_odb10 was built from 11 Poaceae and one Bromeliaceae genome, and Planta et al. (2022) obtained only 72.5-74.4% with it for Carex cristatella, C. scoparia, and two Juncus species, against about 96% with embryophyta_odb10. Please add an embryophyta_odb10 (or odb12) result and this comparison. 

 4. The assembly resolved 27 pseudomolecules, implying n = 27; however, published counts for C. divulsa are higher. The Chromosome Counts Database lists 2n = 58 for subsp. divulsa and 2n = 56 or 58 for subsp. leersii. 2n = 54 was recorded only for the segregate C. chabertii.

 Carex is holocentric, and chromosome fission and fusion produce frequent intraspecific variation; therefore, the difference may be biological. It could equally reflect joined chromosomes. Please discuss the discrepancy and state whether a cytological count was made for this individual or population; Planta et al. (2022) showed that Hi-C scaffold counts and karyotypes can disagree in Carex.

 5. Chromosome 1 (45.71 Mb, 11.8% of the assembly) is almost twice the length of chromosome 2 (23.38 Mb). In Figure 3, the first 30 Mb shows a blocky, chequered contact pattern, with a clear off-diagonal signal to the start of chromosome 3 and a weaker signal to chromosome 8. A thin stripe from the same region also runs across much of the map.

 6. The blob plot (Figure 6) lists 11 ‘no-hit’ sequences totalling 1.76 Mb, which appear to comprise the three organelle sequences and the eight unplaced scaffolds (0.21 Mb). Several have GC = 0.55 and read coverage from approximately 1,000× to over 3,000×, typical of rDNA.

Are sufficient details of methods and materials provided to allow replication by others?

Yes

Is the rationale for creating the dataset(s) clearly described?

Yes

Are the datasets clearly presented in a useable and accessible format?

Yes

Are the protocols appropriate and is the work technically sound?

Yes

Reviewer Expertise:

plant genome bioinformatics

I confirm that I have read this submission and believe that I have an appropriate level of expertise to confirm that it is of an acceptable scientific standard.

# Reviewer report: Reviewer response for version 1

**DOI:** 10.21956/wellcomeopenres.29522.r164649

**Published:** 2026-09-10

**Authors:** Volker P Brendel

**Affiliations:** 1 Department of Biology, Indiana University Foundation, Bloomington, Indiana, USA

The paper describes a contribution within the Darwin Tree of Life project, concerning the genome sequencing of Grey Sedge. All the workflows that were used have been well established within the larger project and are state of the art. There are no gaps - the paper is sound and meets the criteria for indexing.

 There are only a couple of points for consideration:

 1) There is a couple of references to the use of "singularity" containers. First, the current name is "apptainer." Second, it is not clear where these containers are available for download.

 2) The Data Availability section states that the genome will be annotated. I am wondering whether at least a preliminary annotation could be part of this paper? Maybe not RNAseq, but at least spliced alignment of proteins to provide a preliminary assessment of gene models?

Are sufficient details of methods and materials provided to allow replication by others?

Yes

Is the rationale for creating the dataset(s) clearly described?

Yes

Are the datasets clearly presented in a useable and accessible format?

Yes

Are the protocols appropriate and is the work technically sound?

Yes

Reviewer Expertise:

genome informatics

I confirm that I have read this submission and believe that I have an appropriate level of expertise to confirm that it is of an acceptable scientific standard.
